# Role of vitamin D in the pathogenesis of early-onset preeclampsia: a narrative review

**DOI:** 10.3389/fnut.2025.1598691

**Published:** 2025-06-18

**Authors:** Shu Zheng, Shuai Dong, Huimin Shen, Peng Xu, Chang Shu

**Affiliations:** Department of Obstetrics, Obstetrics and Gynaecology Center, The First Hospital of Jilin University, Changchun, China

**Keywords:** vitamin D, early-onset preeclampsia, placental angiogenesis, oxidative stress, immune modulation

## Abstract

Early-onset Preeclampsia (EOPE) is a severe pregnancy complication that poses significant risks to both maternal and fetal health, often leading to fetal growth restriction and maternal morbidity. Despite extensive research, the etiology of EOPE remains unclear, though emerging evidence suggests that vitamin D (VD) may play an important role in placental development and function. Recent studies associate VD deficiency with adverse pregnancy outcomes, including EOPE, through mechanisms such as impaired trophoblast invasion and immune dysregulation at the maternal-fetal interface. This review aimed to synthesize current literature on the role of VD in the pathogenesis of EOPE. We reviewed *in vitro*, *in vivo*, and clinical studies to evaluate the impact of VD on immune modulation, angiogenesis, oxidative stress, and trophoblast migration and invasion in the placenta. This comprehensive review aims to provide insights into how VD deficiency exacerbates placental dysfunction, contributing to the development of EOPE. These insights support the rationale for VD supplementation as a potential preventive strategy and highlight the need for further clinical investigation.

## 1 Introduction

Early-onset preeclampsia (EOPE) is a severe pregnancy complication defined by hypertension and proteinuria before 34 weeks of gestation, affecting approximately 2–8% of pregnancies worldwide (1). It is associated with adverse maternal and fetal outcomes including fetal growth restriction (FGR), preterm birth and increased maternal morbidity. While the exact etiology of EOPE is multifactorial, abnormal placental development is recognized as a central feature (2).

Among the potential upstream contributors, vitamin D (VD) deficiency has emerged as a candidate risk factor. VD is known to influence key biological processes such as trophoblast invasion, immune tolerance, angiogenesis, and oxidative balance—all of which are commonly disrupted in EOPE (3–5). However, the mechanistic role of VD in EOPE remains less thoroughly explored compared to other maternal and placental factors, and the potential for VD-targeted interventions has yet to be fully elucidated.

This review aims to comprehensively examine the current evidence linking VD deficiency to EOPE, with a focus on mechanistic insights. We synthesize findings from *in vitro*, *in vivo*, and clinical studies to evaluate how VD regulates placental function through its effects on trophoblast biology, vascular integrity, immune balance, and oxidative stress. We also discuss the emerging potential of VD supplementation as a modifiable risk factor in EOPE, particularly in high-risk pregnancies. By integrating molecular mechanisms with clinical relevance, this review seeks to bridge existing knowledge gaps and inform future research directions.

## 2 Pathophysiology of early-onset preeclampsia

Preeclampsia (PE) is a complex, multifactorial disorder of pregnancy characterized by new-onset hypertension and proteinuria, typically after 20 weeks of gestation (6). Among its subtypes, EOPE, which occurs before 34 weeks of gestation, is distinguished by greater severity, higher rates of maternal and fetal morbidity, and a closer association with placental dysfunction compared to late-onset preeclampsia (LOPE) (7).

The pathophysiology of EOPE involves a combination of abnormal placentation, dysregulated maternal immune adaptation, oxidative stress, and impaired vascular remodeling (8–10). Abnormal placentation refers to defective development or function of the placenta, often resulting in inadequate nutrient and oxygen delivery to the fetus (11). In a healthy pregnancy, cytotrophoblasts (specialized placental cells) differentiate into extravillous trophoblasts (EVTs). Trophoblast invasion is the process by which these EVTs migrate into the maternal uterine lining, allowing the placenta to anchor securely and interact with maternal tissues (12). A key aspect of this interaction is spiral artery remodeling, during which EVTs contribute to the transformation of maternal uterine spiral arteries from narrow, high-resistance vessels into wider, lower-resistance channels (13). This process is thought to facilitate adequate maternal blood flow to the placenta and, consequently, to the developing fetus. In EOPE, evidence suggests that trophoblast invasion and spiral artery remodeling may be insufficient, which could contribute to persistently high-resistance blood flow, placental hypoperfusion, hypoxia, and increased oxidative stress (14).

Immune dysregulation is an important aspect in the pathogenesis of EOPE (15). This may involve, but is not limited to, altered maternal immune responses to fetal antigens; other contributing factors such as embryo or systemic damage may also play a role (16). Multiple studies have demonstrated that changes in the maternal immune system can contribute to abnormal placentation and the development of EOPE (17). At the molecular level, EOPE is associated with an imbalance between pro-angiogenic and anti-angiogenic factors, such as reduced placental expression of vascular endothelial growth factor (VEGF) and placental growth factor (PlGF), alongside increased levels of soluble fms-like tyrosine kinase-1 (sFlt-1) and endoglin (18, 19). These changes disrupt angiogenesis, further impairing placental vascularization (20). Additionally, abnormal release of inflammatory cytokines [e.g., tumor necrosis factor-*α* (TNF-α), IL-6], heightened activation of the maternal immune system, and insufficient generation of regulatory T cells (Tregs) contribute to a pro-inflammatory environment at the maternal-fetal interface, exacerbating placental dysfunction (21, 22).

EOPE is also characterized by heightened oxidative stress due to the accumulation of reactive oxygen species (ROS) and insufficient antioxidant defenses in the placenta (9, 23). This exacerbates endothelial dysfunction, maternal hypertension, and further restricts fetal growth (24). The combined effects of impaired trophoblast invasion, defective vascular remodeling, angiogenic imbalance, and oxidative injury underlie the unique clinical and pathological features of EOPE (25).

Importantly, while LOPE is often linked to maternal metabolic and cardiovascular risk factors, EOPE is more directly associated with placental pathology and abnormal early pregnancy adaptation (8). This review therefore focuses on placental-associated mechanisms of EOPE, particularly those processes that are potentially modulated by vitamin D—including trophoblast function, angiogenesis, immune regulation, and oxidative stress.

## 3 VD and its association with EOPE

VD exists in two primary forms in humans: vitamin D_2_ (ergocalciferol) and vitamin D_3_ (cholecalciferol), with the latter synthesized endogenously through ultraviolet exposure (26). Vitamin D_3_ is the dominant form involved in human physiology and is the focus of this review. As a fat-soluble vitamin, VD is naturally found in dietary sources such as cod liver oil, fatty fish, mushrooms and egg yolks. Although VD has been traditionally associated with calcium and phosphorus metabolism, it has also been implicated in broader physiological functions including immune regulation, vascular health, and placental development (27–29).

VD metabolism was once believed to occur primarily in the kidneys (30). However, recent studies have revealed that VD is actively metabolized in multiple tissues, including the female reproductive system (31). Both 25-hydroxyvitamin D_3_ (25 (OH)D_3_) and its receptor, VD receptor (VDR), are expressed in various organs, including the uterus, ovaries, fallopian tubes, mammary glands, and placenta (15). The expression of *α*-hydroxylase enzymes in the decidua and placenta during pregnancy further underscores the crucial role of VD at the maternal-fetal interface (15). VD may assist in maintaining healthy placental development and function by regulating calcium transport and immune modulation within the placenta (32).

During healthy pregnancy, maternal serum 25 (OH)D₃ levels typically rise from early to mid-gestation, supporting fetal skeletal development and placental growth (33, 34). However, individuals with EOPE often exhibit significantly lower serum VD levels compared to normotensive pregnancies, with reported deficits of approximately 10–20% (35). Although optimal VD status remains debated, levels below 20 ng/mL are generally considered deficient (36). According to an earlier classification proposed in 2015, serum 25 (OH)D3 levels in healthy pregnant individuals are generally reported to range from 20 to 30 ng/mL (37). Several studies suggest that serum VD levels below this threshold in early pregnancy may be associated with an increased risk of EOPE, likely due to impaired placental adaptation during the first and second trimesters (37, 38).

Numerous meta-analyses, case–control studies, and randomized controlled trials have consistently shown that low maternal vitamin D status is associated with an increased risk of preeclampsia and EOPE, and that vitamin D supplementation may have a protective effect, particularly in high-risk pregnancies. The key clinical evidence is summarized in Table 1.

**Table 1:** Summary of clinical and epidemiological studies linking vitamin D status and risk of PE/EOPE.

Study type	Main finding	References
Meta-analysis	VD deficiency associated with 78% increased risk of PE	(81)
Meta-analysis	Confirmed correlation between low VD and PE across 7 countries	(81)
Case–control	Lower serum VD in EOPE vs. normotensive pregnancy	(82–85)
RCT	VD supplementation improves serum VD, may reduce PE/EOPE risk	(86, 87)

PE, preeclampsia; EOPE, early-onset preeclampsia; RCT, randomized controlled trials; VD, vitamin D.

## 4 Mechanisms involving VD in EOPE

EOPE remains poorly understood. Beyond its traditional role in regulating calcium and phosphorus metabolism, VD influences early placental development and function through multiple biological pathways, including gene expression, immune modulation, angiogenesis, and antioxidant activity (39). Low serum VD levels are associated with abnormal placental implantation and disrupted uterine spiral artery remodeling, leading to impaired angiogenesis and insufficient placental blood supply (40). These pathological processes may exacerbate placental hypoxia and oxidative stress, thereby contributing to the early onset of EOPE (41).

A growing body of research suggests that VD deficiency may promote the onset and progression of EOPE through both direct and indirect mechanisms (39). In early pregnancy, VD is involved in placental immune regulation and trophoblast cell invasion, both of which are essential for ensuring adequate placental blood flow (42). Therefore, further investigation into the role of VD in immune modulation, angiogenesis, oxidative stress, and trophoblast invasion may clarify the pathogenesis of EOPE and provide a theoretical basis for considering VD as a potential preventive strategy. The following sections will explore these key mechanisms in detail, highlighting the specific effects and influences of VD in EOPE.

### 4.1 Role of VD in maternal-fetal immune tolerance

**Figure 1:**
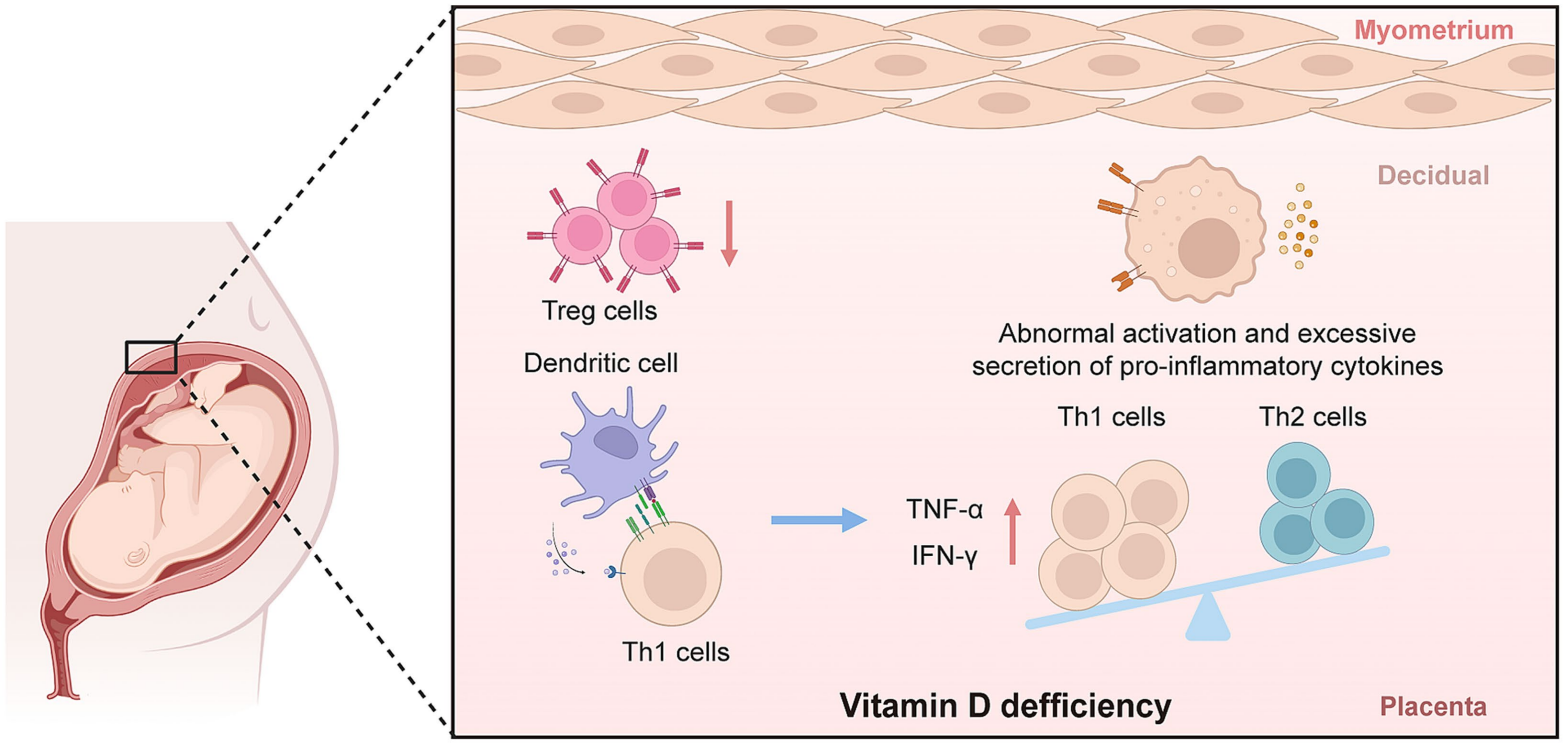
The immunoregulatory role of vitamin D deficiency in EOPE. Created with BioRender and designed by the authors. EOPE, early-onset preeclampsia; IFN-*γ*, interferon gamma; Th, T-helper cells; TNF-α, tumor necrosis factor alpha; Treg, regulatory T cells.

Dysregulation of immune adaptation at the maternal-fetal interface has been widely reported in EOPE. Studies suggest that VD may be involved in the regulation of maternal immune tolerance by promoting Treg function and modulating T helper cell differentiation (43). VD deficiency has therefore been proposed as a potential contributor to placental immune imbalance observed in EOPE (Figure 1).

Experimental and clinical evidence indicates that the active form of VD, 1,25(OH)₂D₃, enhances the expansion and suppressive function of FoxP3 + regulatory Tregs, which are essential for maintaining immune homeostasis at the maternal-fetal interface (15, 44). For example, in patients with EOPE, both peripheral and decidual Treg counts are significantly decreased compared to normotensive pregnant controls, and these alterations have been correlated with lower maternal 25(OH)D₃ concentrations (45, 46). *In vitro* studies using human immune cells have further demonstrated that VD/VDR signaling directly upregulates FoxP3 expression, supporting Treg differentiation and activity (44).

In addition to Treg modulation, VD also influences the Th1/Th2 balance, a key immunological axis in pregnancy. VD has been shown to suppress the production of pro-inflammatory Th1 cytokines, including TNF-*α* and interferon-*γ*, while promoting anti-inflammatory Th2 cytokines such as interleukin-4 (IL-4), interleukin-5 (IL-5), and interleukin-10 (IL-10) (47–50). This effect has been observed in both *in vitro* human T cell studies and clinical cohorts, where VD deficiency is associated with elevated Th1/Th2 ratios and increased placental inflammation in EOPE (46, 50).

Furthermore, studies have demonstrated that VD regulates the activity of dendritic cells (DCs), which play a central role in antigen presentation at the maternal-fetal interface (51, 52). VD inhibits the maturation of DCs and reduces their capacity to activate T cells, thereby limiting local inflammatory responses in the placenta (52, 53). Insufficient VD enhances DC-mediated T cell activation and promotes a pro-inflammatory environment, which has been implicated in abnormal placental development and increased EOPE risk (53, 54).

VD also modulates placental macrophage polarization. VD promotes the M2 anti-inflammatory phenotype while inhibiting the M1 pro-inflammatory phenotype, leading to reduced secretion of TNF-*α* and interleukin-6 (IL-6) in the placenta (55, 56). Both animal models and human studies have linked VD deficiency to increased M1 macrophage infiltration and heightened local inflammation in EOPE placentas (21, 57).

Collectively, these findings from *in vitro*, animal, and clinical studies indicate that adequate VD status supports maternal-fetal immune tolerance by enhancing Treg function, regulating the Th1/Th2 axis, suppressing excessive dendritic cell and macrophage activation, and mitigating placental inflammation. Contrarily, VD deficiency, disrupts these immunological processes, contributing to the immune pathophysiology of EOPE.

### 4.2 Role of VD in reducing impaired uterine spiral artery remodeling

**Figure 2:**
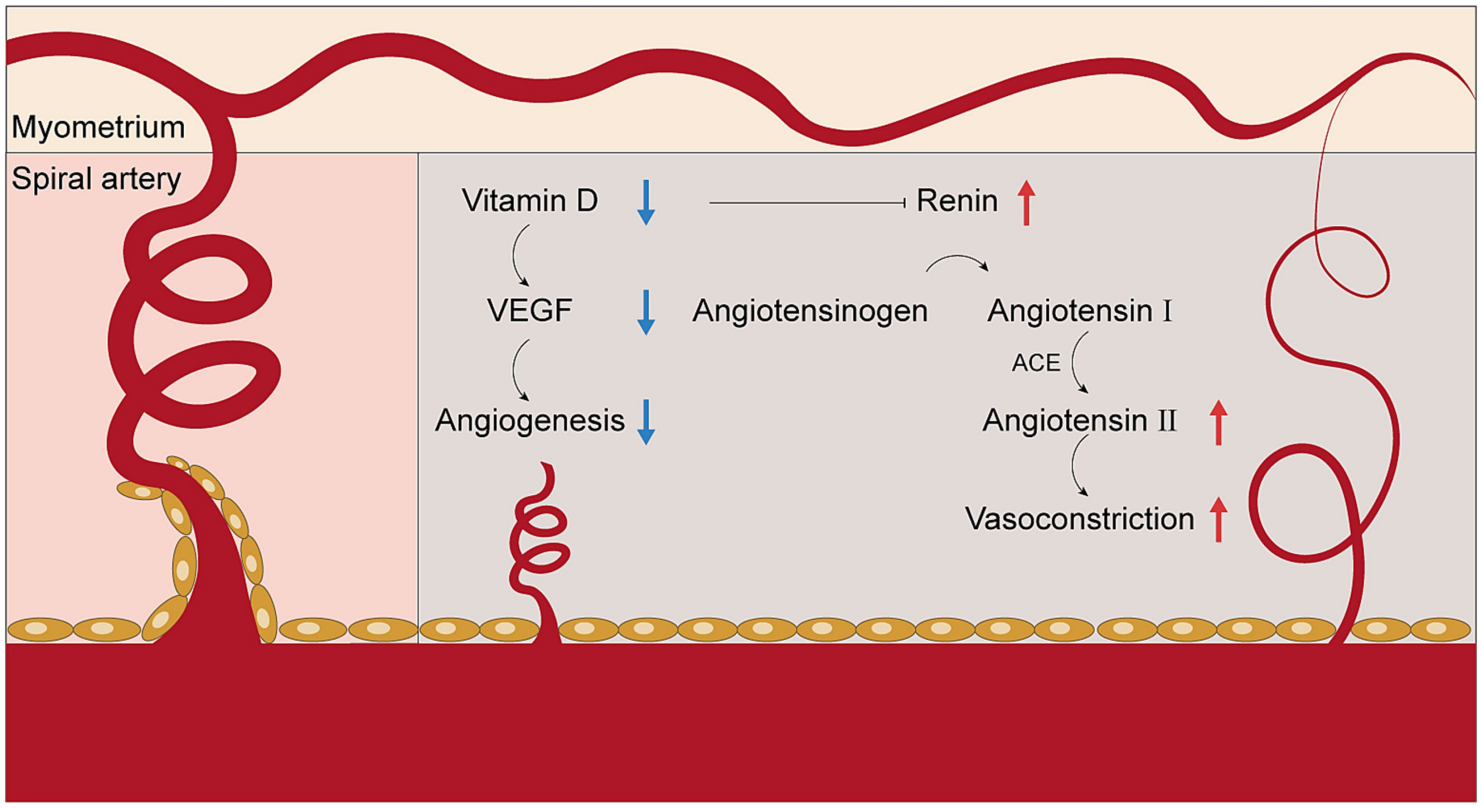
The role of vitamin D deficiency in regulating angiogenesis in the placenta. ACE, angiotensin-converting enzyme; VEGF, vascular endothelial growth factor.

Disruption of placental angiogenesis and inadequate remodeling of the uterine spiral arteries are frequently described features in EOPE (41, 58, 59). Current evidence indicates that VD can influence angiogenic pathways in the placenta, including the regulation of VEGF expression and the renin-angiotensin-aldosterone system (RAAS) (60, 61). The relationship between VD status and placental vascular development remains an area of active research (Figure 2).

Clinical research has corroborated these findings. Analyses of EOPE placental tissue and maternal serum reveal lower levels of VEGF and PlGF, along with elevated concentrations of the anti-angiogenic factor sFlt-1 in women with low VD status (58, 62, 63). These molecular changes correlate with reduced spiral artery remodeling and increased placental vascular resistance, as observed in Doppler ultrasound and histopathology studies.

In addition to directly regulating angiogenic factors, VD is known to modulate the RAAS pathway within the placenta. Experimental animal studies demonstrate that VD suppresses the transcription of the renin gene, leading to lower angiotensin II production and decreased vasoconstriction (61, 64). Clinical data indicate that VD deficiency is associated with increased RAAS activity, contributing to hypertension and further compromising placental perfusion in EOPE (64, 65).

Placental VD receptor (VDR) expression is also reduced in EOPE, which may decrease the placenta’s responsiveness to circulating VD and further limit angiogenic signaling (66–68). Notably, studies report that lower maternal and placental VD/VDR levels are associated with higher risk of FGR secondary to impaired placental blood flow (66, 69).

Although considerable progress has been made in delineating the relationship between vitamin D and placental vascular development, the precise molecular mechanisms—particularly the interplay between VD/VDR signaling, angiogenic factor expression, and RAAS regulation in EOPE—require further investigation in experimental models and large-scale clinical studies.

### 4.3 Role of VD in oxidative stress

**Figure 3:**
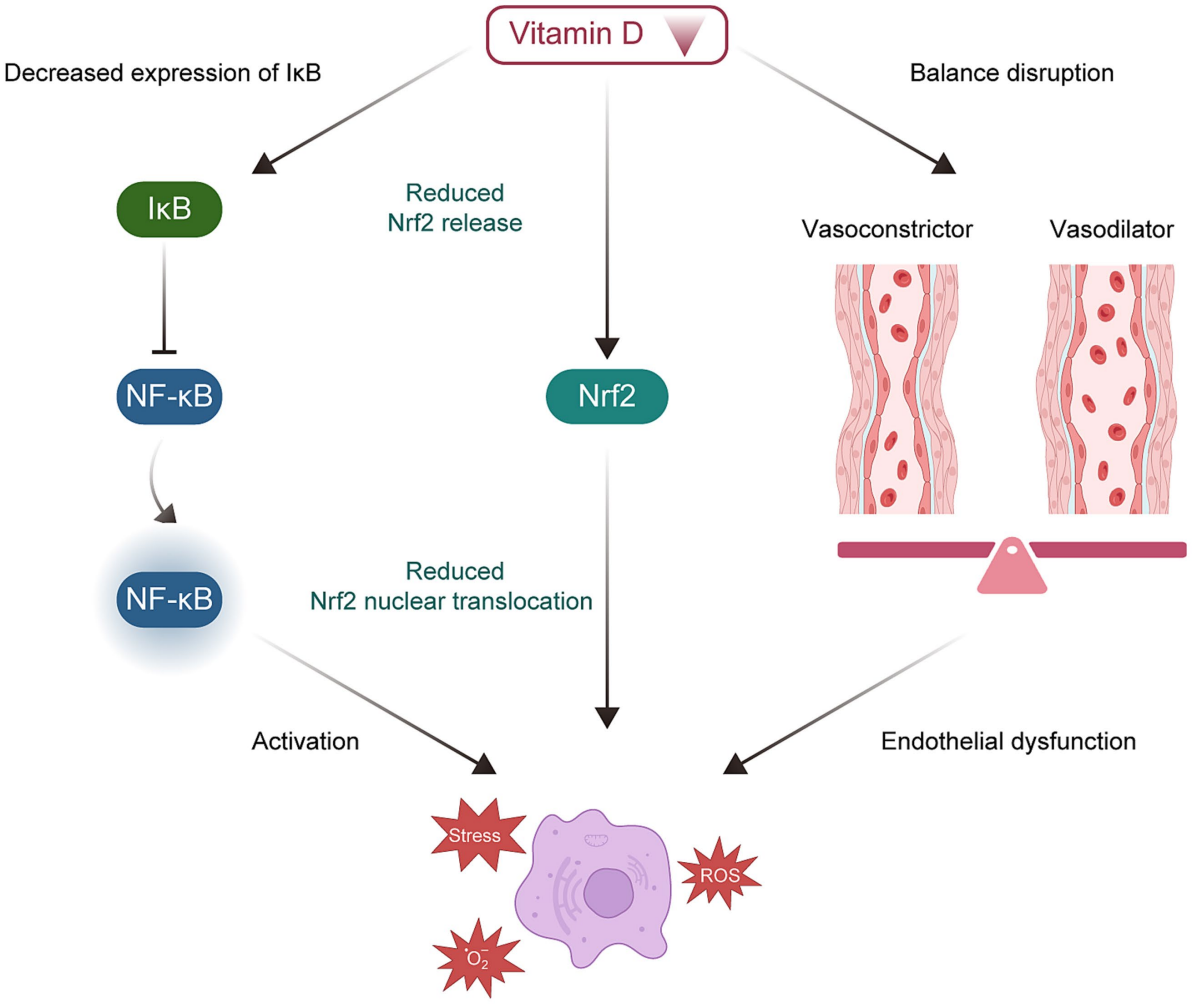
Potential molecular mechanisms of vitamin D deficiency in the development of EOPE. EOPE, early-onset preeclampsia.

Elevated oxidative stress has been implicated in the pathophysiology of EOPE, particularly in relation to placental dysfunction and endothelial injury (23, 70). Experimental and clinical studies have examined the antioxidant properties of VD, including its regulation of key antioxidant enzymes and its effects on oxidative stress pathways (Figure 3) (70–72). The role of VD in modulating placental oxidative stress is being increasingly explored.

*In vitro* studies have shown that 1,25(OH)₂D₃ can upregulate antioxidant enzymes such as superoxide dismutase (SOD) and glutathione peroxidase in placental cells, thereby reducing levels of ROS and lipid peroxidation (70, 73). Consistent with this, women with EOPE and VD deficiency display increased placental malondialdehyde (a marker of oxidative stress) and reduced SOD activity compared to healthy pregnancies (70, 74).

Mechanistically, vitamin D has been reported to inhibit activation of the nuclear factor kappa-light-chain-enhancer of activated B cells pathway in trophoblasts, thereby reducing the expression of pro-inflammatory and pro-oxidant genes and mitigating oxidative injury (70, 72). Furthermore, animal models of preeclampsia have demonstrated that VD supplementation increases nuclear factor erythroid 2-related factor 2 transcriptional activity in the placenta and lowers oxidative stress biomarkers (75).

Although these findings support an antioxidant role for vitamin D in the placenta, the precise molecular mechanisms, especially involving VDR, NF-κB, and downstream effectors such as Nrf2, require further clarification.

### 4.4 Role of VD in EVT migration and invasion

Limited trophoblast invasion and suboptimal remodeling of the maternal uterine arteries have been associated with EOPE in both experimental and clinical observations (76, 77). Research has suggested that VD, via the VDR expressed in trophoblasts, may be involved in the regulation of EVT migration and invasion (78, 79). The possible impact of VD deficiency on these cellular processes is the subject of ongoing investigation.

*In vitro* experiments with human trophoblast cell lines have demonstrated that 1,25(OH)₂D₃ upregulates the expression of matrix metalloproteinases (MMP2 and MMP9), which are essential for extracellular matrix degradation and successful EVT invasion (79). Placental samples from EOPE pregnancies show decreased VDR and MMP9 expression, which are associated with reduced EVT invasive capacity (79, 80). Additionally, vitamin D signaling modulates other molecules involved in cell migration, such as E-cadherin and integrins, which play roles in cell adhesion and motility (59, 78). Importantly, 1,25(OH)₂D₃ stimulates the secretion of human chorionic gonadotropin (hCG) via the cAMP/PKA pathway, which is a well-known regulator of trophoblast motility and invasion (78). Animal studies further indicate that vitamin D deficiency impairs trophoblast invasion and spiral artery remodeling, resulting in phenotypes similar to EOPE (59).

**Figure 4:**
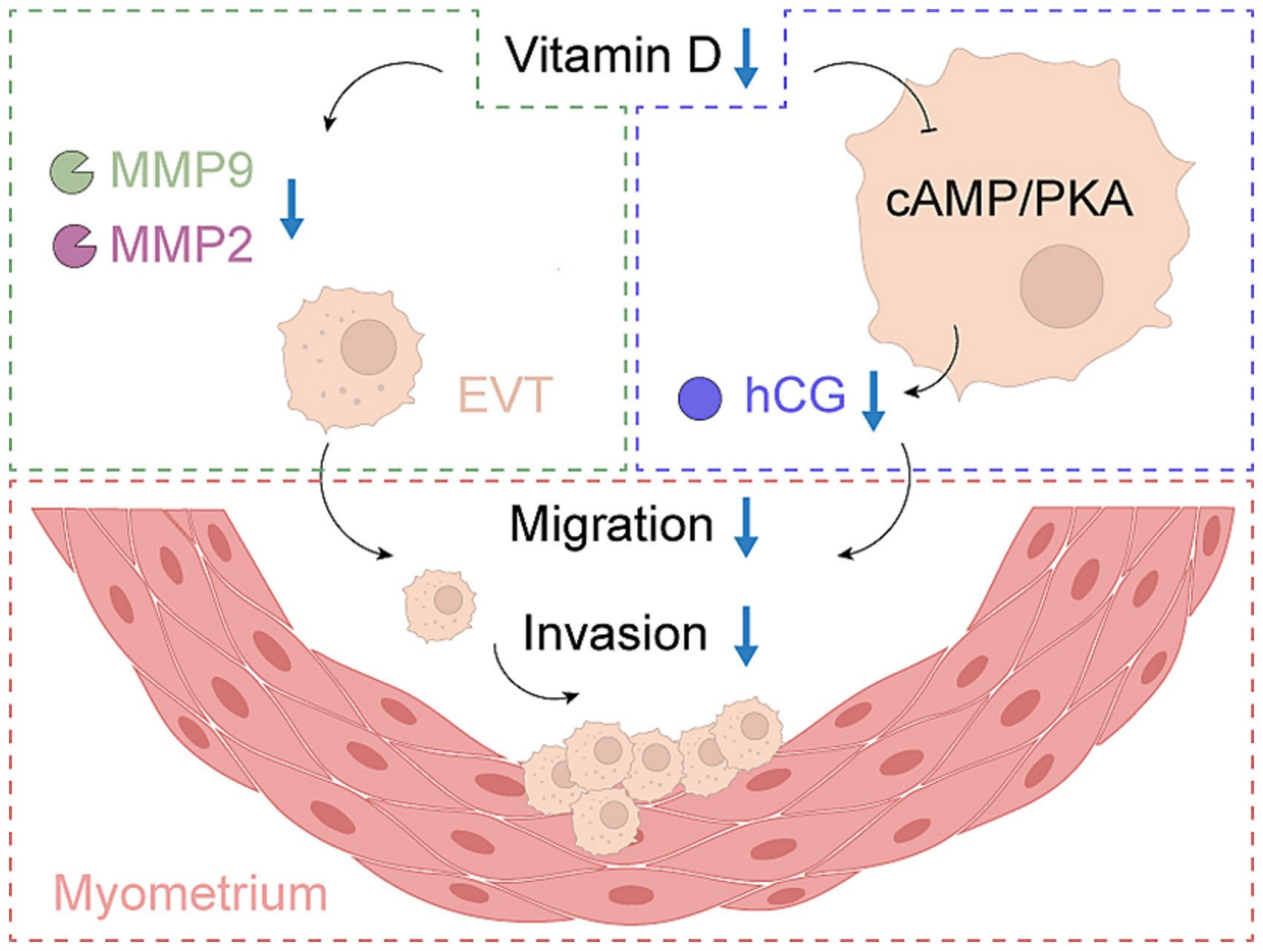
The role of vitamin D in EVT invasion and migration in EOPE. EOPE, early-onset preeclampsia; EVT, extravillous trophoblast; hCG, human chorionic gonadotropin.

Overall, these findings suggest that vitamin D may facilitate EVT migration and invasion by regulating MMPs, adhesion molecules, and hCG-related signaling pathways, but more research is needed to clarify its exact molecular targets in the context of EOPE (Figure 4).

## 5 Conclusion

**Figure 5:**
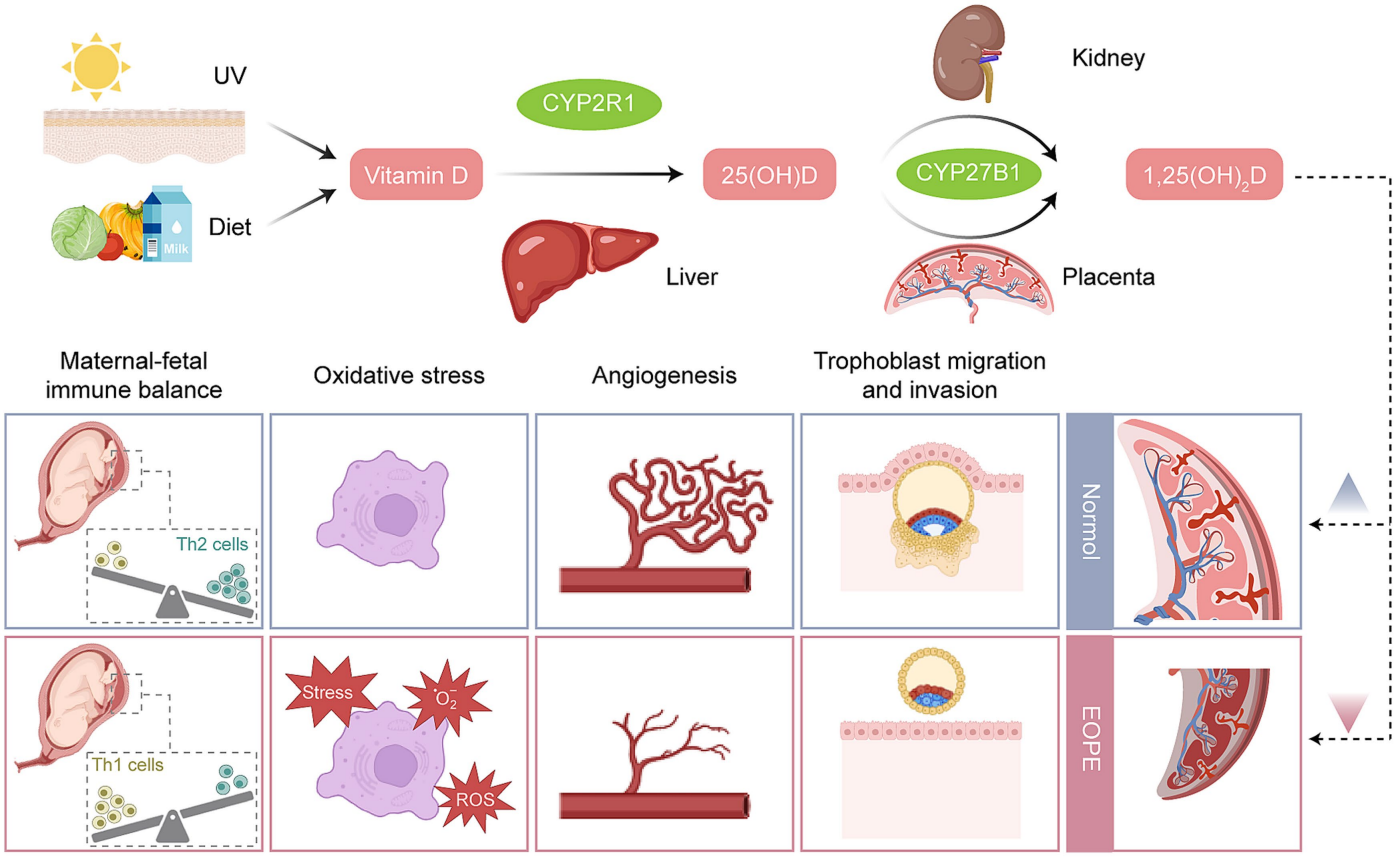
The metabolism of vitamin D and the functional differences in the regulation of placentas between EOPE and normal pregnancy. Created with BioRender and designed by the authors. EOPE, early-onset preeclampsia; ROS, reactive oxygen species; Th cells, T-helper cells; UV, ultraviolet.

VD has been proposed to play a role in the pathogenesis of EOPE, as its deficiency has been associated with impaired placental development, increased oxidative stress, and immune dysregulation at the maternal-fetal interface (Figure 5). Findings from individual studies suggest that VD may influence processes such as angiogenesis and vascular remodeling, which are considered important for supporting healthy pregnancy outcomes. Low VD levels during pregnancy have been associated with an increased risk of EOPE and FGR, and VD supplementation has been proposed as a potential area for therapeutic exploration. Understanding the molecular mechanisms through which VD influences EOPE offers a promising approach to clinical management and prevention. In clinical practice, monitoring and managing VD levels has been suggested as a potentially beneficial approach, especially in high-risk pregnancies.
